# The Regional Distribution of Psychiatric Residency Positions Funded by the Department of Veterans Affairs and Its Relationship to Rural Veteran Populations

**DOI:** 10.1007/s40596-021-01565-1

**Published:** 2021-11-17

**Authors:** Matthew Vincenti, Anthony Albanese, Edward Bope, Bradley V. Watts

**Affiliations:** 1 Veterans Rural Health Resource Center, White River Junction, VT USA; 2 Geisel School of Medicine at Dartmouth, Hanover, NH USA; 3 U.S. Department of Veterans Affairs’ Office of Academic Affiliations, Washington, DC USA

**Keywords:** Internship and residency, Healthcare sector, Training support, Psychiatry, Rural population

## Abstract

**Objective:**

The authors evaluated the distribution of psychiatry residency positions funded by the Department of Veterans Affairs between 2014 and 2020 with respect to geographic location and hospital patient population rurality.

**Methods:**

The authors collected data on psychiatry residency positions from the Veterans Affairs’ Office of Academic Affiliations Support Center and data on hospital-level patient rurality from the Veterans Health Administration Support Service Center. They examined the chronological and geospatial relationships between the number of residency positions deployed and the size of the rural patient populations served.

**Results:**

Between 2014 and 2020, the Department of Veterans Affairs has substantially increased the number of rural hospitals hosting psychiatry residency programs, as well as the number of residency positions at those hospitals. However, several geographic regions serve high numbers of rural veterans with few or no psychiatry resident positions.

**Conclusions:**

While the VA efforts to increase psychiatry residency positions in rural areas have been partially successful, additional progress can be made increasing support for psychiatry trainees at Veterans Affairs hospitals and community-based outpatient clinics that serve large portions of the rural veteran population.

The Department of Veterans Affairs (VA) operates the USA’s largest integrated healthcare system, providing care at 171 hospitals and 1112 outpatient sites to more than 9 million veterans [1]. The VA funds more than 10,000 residency positions in more than 80 specialties across more than 2600 graduate medical education programs [2, 3] supporting more than 30% of all resident positions in the USA.

Recent studies suggest that provider shortages in rural areas are currently problematic and likely to increase dramatically in the coming years, due to an aging provider demographic [4] and declines in rural medical student applicants, who are more likely to practice in rural areas [5]. Rural psychiatry needs suffer disproportionate provider shortages compared to urban areas. The reasons for rural psychiatry workforce shortages are multifactorial but are believed to be linked to fewer clinicians being trained and initiating practice in a socioeconomically challenged and a resource-restricted clinical environment [6]. Therefore, increasing psychiatry resident training programs and residency positions in rural areas has been posited as a potential solution for rural psychiatry workforce shortages and the resultant mental health disparities found among rural populations [7]. As a funder of nearly a third of all residency positions, VA support of rural residency programs is essential to increase the number of psychiatry residents in rural areas.

The VA Office of Academic Affiliations provides salary support for residency positions occurring at VA hospitals [1]. This salary support is typically directed from individual VA hospitals to their partnered academic institutions who administer the residency program. In this way, the VA funds some number of residency positions at most graduate medical education sites in the USA. The *Veterans Access, Choice, and Accountability Act* of 2014 [1] provides funding to expand the number of residents who do clinical rotations at VA hospitals. This funding has allowed many residency programs to expand their number of residents. In addition, the VA Office of Academic Affiliations has provided grants for graduate medical education expansion planning and educational infrastructure support.

In accordance with the *Veterans Access, Choice, and Accountability Act* of 2014, the VA has a goal of funding 1500 additional residency positions nationwide by 2024 but has acknowledged challenges creating positions at rural sites [8]. While the VA has prioritized providing psychiatry resident positions at rural sites, many rural veterans receive their care at urban VA Medical Centers and increasingly through telehealth. Therefore, resident positions deployed by the VA at some urban facilities are also essential for providing healthcare to veterans in rural regions. VA medical facilities across the country are administered by eighteen regions with each region comprised multiple healthcare markets. Thus, an analysis that compares the national distribution of funded resident positions and rural VA patients they serve seems warranted. In this study, the authors evaluated the distribution of psychiatry residency positions funded by the VA between 2014 and 2020 with respect to geographic location and the population of rural patients served.

## Methods

### Data Sources

The VA compiles data annually for all residency positions funded at participating VA hospitals across the country. Aggregated data on psychiatry residencies for 2014 through 2020 were accessed from the Office of Academic Affiliations Support Center. VA hospital data, comprised hospital name, number, and rurality of patients, was obtained from the VA Support Service Center [9]. Patient-level rurality data was obtained from the VA Support Service Center Patient Enrollment Cube, combining the number of rural patients who used a hospital participating in an Office of Academic Affiliations–funded Psychiatry residency program each year between FY2014 and FY2020. All years represent end of year totals except for 2020, which represents totals as of September 4, 2020. Rurality was defined using rural–urban commuting area categorizations of US census tracts [10]. Census tracts are categorized as metropolitan, micropolitan, small towns, and rural areas. Census tracts are further defined by the percentage of residents who commute to and from urbanized areas or urban clusters. For this study, urban census tracts were designated as those residing in metropolitan areas and with 30 to 50% commuter flow to a larger urbanized area. All other census tracts were designated as rural. The VA also collects data on insular island veterans, but these veterans were not included in the rurality calculation. Datasets were imported to JMP Pro 15 and joined by matching year and VA facility number.

### Data Presentation

The psychiatry resident and veteran patient data represent entire populations for a given year so that any differences are considered significant. Tables were created by summing the number of yearly psychiatry resident positions funded and then stratifying by rural hospital, hospital rurality, VA program (“Traditional” versus “Expanded” allocations), and VA-defined healthcare markets. Numbers of psychiatry residents shown represent the number of full-time employee equivalents rounded to whole numbers for clarity. The total number of resident positions allocated to each VA-defined region between 2014 and 2020 was divided by the total number of VA patients in that region during the time period and then multiplied by 10,000 (“Resident Positions Per Ten Thousand Patients”). The “Percentage of Rural Patients” was calculated by dividing the number of rural patients by the number of total patients in each region. Four categories were created by binning values above the median (high) and below the median (low) for both the “Resident Positions Per Ten Thousand Patients” and “Percentage of Rural Patients” values. A choropleth was generated by mapping healthcare markets assigned to one of the four categories (High High, High Low, Low High, and Low Low).

## Results

To assess the impact of VA support for psychiatry residency programs in rural areas, we examined the number and geographic distribution of VA-funded residency training sites. Between 2014 and 2020, 11 of the 25 VA hospitals in rural locations received psychiatry resident position support from the VA (Table 1). The fourteen rural VA facilities that did not receive psychiatry resident positions were in Bath, NY; Batavia, NY; New Castle Road, PA; Carrolton, GA; Lake City, FL; Iron Mountain, MI; Marion, IL; Tuskegee, AL; Poplar Bluff, MO; Bonham, TX; Kerrville, TX; Hot Springs, SD; Sheridan, WY; and Fort Harrison, MT. During this period, the number of rural VA hospitals with any psychiatry residency positions increased from 5 in 2014 to 10 in 2020. In 2020, Dublin, GA did not receive any resident positions. Therefore, for that year, only 10 of the 11 rural sites had positions (Table 1). Paralleling the increase in rural sites, the number of supported psychiatry positions at those rural sites increased from 20 to 52 (Table 2), a 160% increase. By comparison, participating urban sites increased from 96 to 103 and psychiatry resident positions at urban sites increased from 978 to 1131 (Table 2), a 15.6% increase. The total number of positions was not evenly distributed among the rural facilities supported, with Dublin, Fort Mead, and Roseburg receiving relatively few positions (Table 1). Thus, while rural psychiatry residency support increased between 2014 and 2020 and was proportionally greater than at urban sites, not all rural sites were engaged and not all rural sites were supported similarly.

**Table 1:** Psychiatry residency position allocation at rural Veterans Affairs medical centers. The number of psychiatry resident positions supported by the Department of Veterans Affairs between 2014 and 2020 are presented with total positions by facility and by year

Residency location	2014	2015	2016	2017	2018	2019	2020	Total positions by location
Big Spring, TX	0	0	4	4	4	4	4	20
Chillicothe, OH	7	8	9	8	8	9	8	57
Clarksburg, WV	1	1	1	1	1	1	1	7
Dublin, GA	0	0	0	0	1	0	0	1
Fort Meade, SD	0	0	0	1	0	1	1	3
Leavenworth, KS	3	2	3	3	3	13	14	41
Muskogee, OK	0	0	4	5	5	8	5	27
Roseburg, OR	0	0	0	1	1	1	1	4
Togus, ME	2	2	1	2	2	1	2	12
Tomah, WI	0	0	0	1	1	2	3	7
White River Junction, VT	7	7	4	5	5	5	6	39
Total positions by year	20	20	26	31	31	45	45

**Table 2:** Relative distribution of psychiatry resident positions at rural and urban Veterans Affairs medical centers. The total number of psychiatry resident positions supported between 2014 and 2020, by the Expanded Resident Allocations due to federal legislation and by the Traditional Resident Allocations, is shown

Year	Expanded resident allocations		Traditional resident allocation		Annual percentage of rural residents
	Rural residents	Urban residents	Rural residents	Urban residents	
2014	0	0	20	978	2.03%
2015	0	36	20	946	1.96%
2016	6	62	24	968	2.83%
2017	10	88	25	955	3.18%
2018	9	114	25	968	3.03%
2019	11	167	38	942	4.16%
2020	17	212	36	919	4.45%

When examining the effect of the new funding provided by the Veterans Access, Choice and Accountability Act of 2014, several trends emerged. These “Expanded Resident Allocations” supplemented “Traditional Resident Allocations,” starting in 2015 for urban sites and 2016 for rural sites (Table 2). Expanded resident allocations increased annually at both rural and urban sites, with the vast majority supporting urban sites. For each year examined, the number of expanded resident allocations at rural sites was typically less than 10% of expanded resident allocations at urban sites. However, each year, the proportion of expanded resident allocations to total resident allocations was consistently higher at rural sites.

**Fig. 1:**
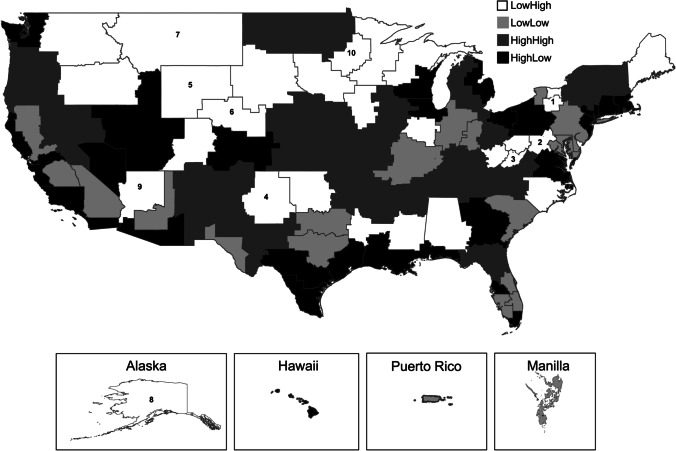
Relationship between psychiatry resident positions deployed and rural patient populations in the Department of Veterans Affairs–defined healthcare markets. The number of psychiatry positions per ten thousand patients and the percentage of rural patients were calculated for all healthcare markets. Markets were then stratified as above the median (High) or below the median (Low) for the two measures. White markets numbered one through ten correspond to those markets (Table 3) with no resident positions allocated

To better understand the impact of VA-funded psychiatry residency positions, we compared the population-adjusted number of VA-funded psychiatry residency positions to the density of rural VA patients in each of the previously defined 98 VA regions [11]. This analysis demonstrated that regions with relatively high numbers of psychiatry residency positions and high numbers of rural veterans are dispersed across all regions of the country (Fig. 1, dark grey markets). This includes markets served by VA medical centers in urban centers such as Memphis, Fargo, Charleston, Nashville, Lexington, Kansas City, Albuquerque, Omaha, Albany, Gainesville, Ann Arbor, Little Rock, Iowa City, and Portland. However, markets with relatively low numbers of residency positions and high numbers of rural veterans (Fig. 1, white markets) reside in the Northeast, Southeast, Midwest, and Northwest. Of concern, ten markets received no support for psychiatry residency positions, with most of these markets serving greater than 50% rural veterans (Fig. 1, Table 3). In stark contrast to these under-resourced and rural markets, relatively high numbers of psychiatry residency positions have been deployed at markets containing urban centers serving few rural veterans, such as New York, Boston, Pittsburg, Washington D.C., Atlanta, Chicago, Denver, Houston, Salt Lake City, San Francisco, San Diego, and Los Angeles (Fig. 1, black markets). These urban facilities serve high numbers of veterans, so the high number of residents might be warranted based on patient panel sizes. However, a more detailed analysis of markets suggests an urban bias in resident funding. A regression analysis demonstrated a reduction in the number of residents per total number of patients (both rural and urban) with increasing percentages of rural patients in each region (slope =  − 2.93, *F* statistic = 8.11, *P* = 0.005). This regional analysis of VA healthcare region demonstrates that rural service areas are supported by a paucity of psychiatric trainees at VA hospitals.

**Table 3:** Identification of healthcare markets with low resident allotments and large rural populations. The twenty-seven healthcare markets serving high percentages of rural patients and allotted little or no psychiatry resident positions per ten thousand patients are shown

Market	Resident positions per ten thousand patients	Percentage of rural patients
1	0.00	78%
2	0.00	50%
3	0.00	66%
4	0.00	48%
5	0.00	80%
6	0.00	34%
7	0.00	71%
8	0.00	49%
9	0.00	52%
10	0.00	76%
11	0.63	63%
12	0.75	66%
13	0.76	87%
14	0.78	80%
15	0.95	32%
16	1.00	43%
17	1.07	48%
18	1.25	33%
19	1.44	81%
20	1.47	52%
21	1.48	42%
22	1.53	38%
23	1.63	69%
24	1.67	44%
25	1.83	39%
26	1.93	57%
27	2.17	47%

## Discussion

We found growth in the number of psychiatry residency training sites, as well as the number of psychiatry resident positions, funded by the VA at rural locations between 2014 and 2020. In addition, our regional analysis highlighted that several residency programs in urban areas are serving high numbers of rural veterans. However, we also found that several markets with the highest percentages of rural patients received the fewest number of psychiatry resident positions per capita. These regional scarcities of psychiatric positions can have profound implications for rural workforce development and access to care.

Currently, there is a dire need for additional psychiatrists in rural areas and expansion of rural residency opportunities can help meet this need [12]. While growing up in a rural area is the strongest predictor of practicing in a rural area, training at a rural site has a moderate effect on providers choosing a rural practice [13]. Thus, VA-funded residencies may help recruit more psychiatrists to rural practice as well as address immediate rural provider shortages. Our analysis demonstrates that the VA through its Office of Academic Affiliations has made substantial progress through establishing additional psychiatry training sites in rural areas. Due to limited staffing and lower clinical complexities, residencies in rural locations are challenging to establish and maintain. Currently, the VA only supports new residency programs that are affiliated with an existing academic program. Thus, part of the challenge is that there are rural areas with many patients but no existing psychiatry residency program for the VA to partner with and therefore no ability to expand residency positions. This highlights the potential for the VA to partner with other governmental agencies and new academic partners to develop new psychiatry training programs in these rural regions. Recent federal legislation [8] offers two new options to enhance recruitment of physicians (including psychiatrists) into rural areas. These options include a Health Professional Scholarship Program for medical students, and a Specialty Loan Repayment Program, both with payback at locations approved by the VA. Another section of this legislation includes a pilot program, placing not less than 100 residents in Indian Health Service or tribal healthcare facilities, or designated underserved VA areas. This pilot will include educational infrastructure support for selected new residency programs as well.

Urban sites also play an important role, and it is essential to understand the rural impact of training programs at urban hospitals. Our work suggest that additional progress can be made through increasing and focusing graduate medical education expansion toward rural VA facilities and regions of the country with larger portions of the rural veterans. This strategy could be enhanced by advocating for more clinical training resources and greater investment in satellite clinics and supporting telepsychiatry.

A limitation of our analysis is that we were only able to collect data down to the level of major medical centers. Although these medical centers also provide care through community-based outpatient clinics, we were unable to report on the contributions of these satellite facilities for resident training and rural veteran care. While this study suggests impact of these initial efforts to enhance the rural psychiatry workforce through funding of new psychiatry resident positions at rural sites, additional work will be required to determine if these efforts result in a long-term enhancement in the rural psychiatry workforce.
